# Modulation of Bax and Bcl-2 genes by secondary metabolites produced by *Penicillium rubens* JGIPR9 causes the apoptosis of cancer cell lines

**DOI:** 10.1080/21501203.2019.1707315

**Published:** 2019-12-26

**Authors:** Prerana Venkatachalam, Varalakshmi Kilingar Nadumane

**Affiliations:** Department of Biotechnology, School of Sciences, JAIN (Deemed-to-be University), Bengaluru, India

**Keywords:** Cytotoxicity, caspase activities, cell cycle, Bax expression, secondary metabolites, *Penicillium rubens*

## Abstract

Search for an efficient anti-cancer compound of natural origin with well-defined mechanisms of action is an important scientific pursuit today, due to cancer being the second leading cause for the death of affected people. The members of the genus *Penicillium* are one of the important sources of bioactive compounds. In the present study, *Penicillium rubens*, isolated from a garden soil in Madurai district of Tamil Nadu, was found to produce a highly promising anti-cancer metabolite. The percentage viabilities of HepG2, HeLa and MCF-7 cancer cells treated with the bioactive fraction (P5) isolated from *P. rubens*, ranged between 40-50% after 96 h. Apoptosis induction was found to be the major reason for the observed reduction in cancer cell proliferation and cell count which was confirmed by caspase activity, DNA fragmentation, clonogenic assay, cell cycle analysis and LDH assays. The upregulation of proapoptotic Bax, coupled with the downregulation of anti-apoptotic Bcl-2 expressions were confirmed by RT-qPCR and flow cytometry methods. The current study also indicated an upregulation of p53 which further strengthened the apoptogenic property of P5 fraction. Non-toxicity of P5 was demonstrated on normal peripheral lymphocytes. The analysis of P5 fraction through GC-MS indicated the presence of indole-2, 3-(4,4-dimethyl-3-thiosemicarbazone) as one of the major compounds.

## Introduction

Cancer is a dreadful disease affecting a large percentage of humans and despite advanced treatment modalities with the death rate being very high, the goal for a successful cancer therapy demands the identification of highly efficient therapeutic molecules. The high toxicity and side effects associated with anti-cancer drugs increases the need for novel drugs active against diverse kinds of tumours, with lesser or no side effects (Demain and Sanchez 2009). Nature is considered to be one of the most important sources for pharmacologically active compounds as part of drug discovery (da Rocha et al. 2001; Bhatnagar and Kim 2010). Natural products have played a significant role in the treatment of cancer over the past few decades.

Filamentous fungi have gained increased attention and are being exploited extensively as they are the producers of novel secondary metabolites of therapeutic significance which have proven as superior to products of chemical origin and have shown their usefulness as anti-cancer agents (Calvo et al. 2002; Kinghorn et al. 2016). *Penicillium* sp. are a class of fungi that are widely distributed in nature producing secondary metabolites of pharmaceutical importance such as the β-lactum antibiotics penicillin, xanthocillins, sorbicillin, chrysogine among many others (De Hoog et al. 2000; Mohammad et al. 2011). The members of the fungal genus *Penicillium* have been utilised worldwide for the production of highly versatile cytotoxic secondary metabolites. Many of the metabolites isolated from various species of the genus *Penicillium* have shown promising growth-inhibitory properties against different *in-vitro* as well as *in-vivo* human cancers (Kornienko et al. 2015; Koul and Singh 2017; Youssef and Alahdal 2018). Several lead compounds from terrestrial fungi are currently being used for medicinal purposes (Gomes et al. 2015). It has been estimated that only around 100,000 species out of the approximately 3 million fungal species on Earth have been described so far. Therefore, to find an efficient anti-cancer compound from fungal source, which is also safe to the normal healthy cells, filamentous fungi were isolated from different sources and screened for their cytotoxicity to *in-vitro* cancer cell lines. Based on the results of initial screening, among the isolates, *Penicillium rubens* JGIPR9 was chosen for the current study.

## Methods

### Isolation of fungi

Filamentous fungi were isolated from many environmental sources (air, water, soil and phylloplane) by serial dilution method (Aneja 2003). The organisms were isolated on M9 medium, supplemented with 1% casein. Different coloured fungi were selected and screened for their cytotoxicity to cancer cell lines. The fungal cultures were maintained in Czepak dox yeast agar. The isolate with the highest cytotoxic effect was chosen and identified by molecular methods.

### Extraction of secondary metabolites

A primary inoculum containing 2×10^6^ fungal spores/ml was used for the culture studies. The chosen fungus was incubated in Czepak Dox Yeast Broth at 24-28°C under static conditions for 12 days. The mycelium was removed by filtering through Whatmann filter paper and was dried overnight at 60°C. The dried biomass was later homogenised and the metabolites were extracted using methanol. The extract was evaporated to dryness and the dried extract was used for initial cytotoxicity screening.

### Cell lines

Cancer cell lines MCF-7, HeLa and HepG2 were acquired from NCCS, Pune, India. Healthy human peripheral lymphocytes were used as control cells. Cells were maintained in MEM (HiMedia) supplemented with 10% FBS (HiMedia) and incubated at 37°C with 5% CO_2_.

### Isolation of lymphocytes

Isolation of lymphocytes from blood was performed as per the ethical guidelines outlaid by Indian Council of Medical Research (ICMR) (Mathur 2017) following a standard protocol (HiSep LSM 1077, HiMedia) with the addition of phytohaemagglutinin-L (Himedia) to stimulate proliferation.

### MTT assay

Cells were seeded at an initial concentration of 1×10^4^ cells/ml onto 96-well microtiter plates. After 24 h, the methanol extract from the fungal biomass was added at varying concentrations (1, 10, 50 and 100 µg/ml) for 24, 48, 72 and 96 h. The cytotoxic effect was assessed by the addition of MTT dye as per the standard protocol (Mosmann 1983).

### Fractionation by preparative thin layer chromatography (TLC)

The methanol extract was partially purified by preparative TLC using Silica gel 60 F_254_ (Merck) as previously reported (Rabel and Sherma 2017). Each of the partially purified fraction was screened for its cytotoxicity on cancer cells and the one with highest activity was chosen for further studies.

### Morphological observation

Morphological changes after 48 h of cancer cells (2x10^6^ cells/ml) treated with sample (25 μg/ml) along with the untreated control were observed under an inverted microscope (Labomed, Germany) (Freshney 2000).

### Fluorescence microscopy

Cancer cells (2x10^6^ cells/ml) and healthy lymphocytes were treated with the sample (25 μg/ml) for 48 h along with untreated control. The cells were stained with acridine orange/ethidium bromide (AO/EtBr) and observed under the fluorescence microscope (Brousseau et al. 1999).

### DNA fragmentation analysis

Cancer cells (2x10^6^ cells/ml) were treated with the sample (25 μg/ml) for 48 h along with untreated control. The DNA from untreated and treated cancer cells were isolated as per the previous methodology (Gavrieli et al. 1992) and were electrophoresed on 1% agarose gel along with 1kb ladder DNA (Genei).

### Lactate dehydrogenase (LDH) activity assay

The LDH activity of the cancer cells (2x10^6^ cells/ml) treated with the sample (25 μg/ml) for 48 h was determined as per the kit manufacturer’s instructions (G-Biosciences, cat. # 786–210) (F K-M et al. 2013).

### Caspase −3, −7 and −10 activities

Cancer cells (2x10^6^ cells/ml) were treated with the sample (25 μg/ml) for 48 h along with untreated control. The activities of caspase −3, −7 and −10 were analysed as per the kit manufacturer’s instructions (G-Biosciences, cat. # 786–202 B) (Béchohra et al. 2016).

### Nitric oxide (NO) assay

The NO release in cancer cells and healthy lymphocytes (2x10^6^ cells/ml) treated with the sample (25 μg/ml) for 48 h was estimated using standard protocol (Ding et al. 1998). The nitrite concentration was expressed as µM/ml.

### Clonogenic assay

The ability of cancer cells to form colonies after sample treatment for 48 h at 25 and 50 µg/ml concentrations were assessed by performing clonogenic assay as per the standard protocol (Franken et al. 2006). The cells were harvested by trypsinisation, washed with PBS and immediately seeded onto culture flasks at very low concentrations (100–200 cells/ml). The colony formation was observed after a period of 14 days. The colonies formed were washed with phosphate buffer, fixed in acetic acid and methanol fixative for 30 min followed by staining with crystal violet (1%). The number of colonies was determined by counting the colonies with >50 cells as the minimum size for a colony.

### Cell cycle analysis

Stages of cell cycle were analysed for DNA content using a BD FACS Verse flow cytometer (IISc, Bengaluru) after treating the cancer cells (2x10^6^ cells/ml) with the sample (25 μg/ml) for 48 h (UC San Diego Health Sciences Protocol – https://medschool.ucsd.edu/research/moores/shared-resources/flow-cytometry/protocols/Pages/cell-cycle-with-pi.aspx). The percentage of cell populations in sub-G1, G1, S and G2 phases was estimated using FACSDiva version 6.1.3.

### Analysis of Bcl-2, Bax and p53 expressions at the mRNA and protein levels

The anti-apoptotic Bcl-2, pro-apoptotic Bax and tumour suppressor p53 expressions at the mRNA level were assessed by RT-qPCR and at the protein level by flow cytometry as per the previous methodology (Venkatachalam and Nadumane 2018).

### Gas chromatography – mass spectrometry (GC-MS) analysis

The GC-MS analysis was carried out for the fraction P5 at The South Indian Textile Research Association (SITRA), Coimbatore. The analysis was performed with a Thermo GC-Trace Ultra Ver: 5.0, Thermo MS DSQ II in DB 35 – MS Capillary standard non-polar column, dimensions: 30 m (L) x 0.25 mm (ID) x 0.25 µm film thickness. The carrier gas used was Helium and the flow rate was 1 ml/min. 1 µl of the sample was injected to the column. The initial temperature for the program was 70°C raised to 260°C at 6°C/min, total time was 37.50 min. Scan mass range was 50–650 m/z. The spectrum of the unknown compound was analysed using an online database for anti-cancer molecules (Cancer Resource Database – http://data-analysis.charite.de/care/).

### Statistical analysis

All of the experiments were performed thrice and the results were expressed as mean ± standard error. Significance of the results were checked by one-way/two-way ANOVA (GraphPad Prism 6.0 software). A *p*-value corresponding to <0.05 was taken for reporting statistical significance.

## Results

### Identification of the organism

Out of the 35 fungal isolates, the one which was isolated from garden soil obtained from Madurai district, Tamil Nadu was exhibiting promising cytotoxic and anti-cancer properties. The isolate was identified as *Penicillium rubens* JGIPR9 (Accession no. MH816935) by performing 18 s rRNA sequencing (Biokart India Pvt. Ltd., Bengaluru).

### Evaluation of cytotoxicity

**Figure 1.**
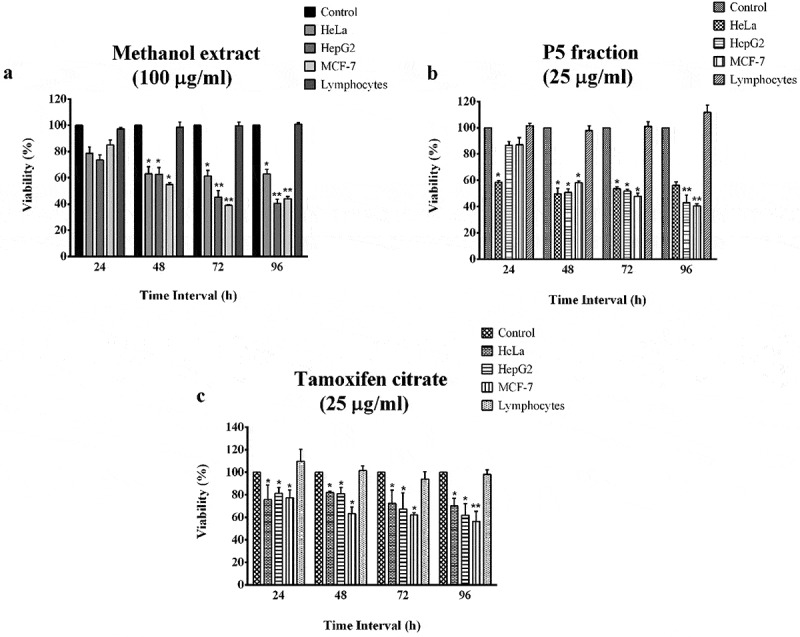
Anti-cancer effect of the methanol extract and P5 fraction from *Penicillium rubens*along with tamoxifen citrate as positive control. *p< 0.05 and **p< 0.01 indicate the levels of significance in comparison to the control

When different concentrations of the methanol extract from the fungal isolate were checked for its cytotoxicity against cancer cells, significant cytotoxic effect was observed (Figure 1(a)). The highest cytotoxic effect was on MCF-7 cells at 100 µg/ml concentration after 72 h of treatment with a viability of 39.09%, followed by HepG2 cells with 40.54% and HeLa cells with 60.34%, both after 96 h treatment.

When the methanol extract was subjected to preparative TLC for fractionation, ethyl acetate and acetone (1:1) used as the solvent system resulted in separating five fractions (Bands P1 – P5). The cytotoxic effect of all the fractions were tested against the cancer cell lines and band 5 (P5) was found as the one with highest effects (results not shown). This fraction was checked for its anti-proliferative effects at 1, 10 and 25 µg/ml concentrations. The cytotoxic effect was significant at 25 µg/ml concentration with a viability of 49.74% in HeLa cells after 48 h, 42.92% in HepG2 cells after 96 h and 40.49% in MCF-7 cells after 96 h (Figure 1(b)). The IC_50_ values were calculated as 24.4 µg/ml for HepG2, 11.0 µg/ml for HeLa and 16.0 µg/ml for MCF-7 cells. The viability of lymphocytes was found to be higher than 98% at all the tested concentrations even after 96 h of incubation period with both crude and TLC purified fractions (Figure 1 (a,b)).

When the cytotoxic effect of tamoxifen citrate, the positive control, was tested on cancer cell lines and lymphocytes, there was a significant cytotoxic effect with 62.1% viability in MCF-7 cells, followed by 67.2% and 72.14% in HepG2 and HeLa cells, respectively, after 72 h at 25 µg/ml treatment concentration. The viability further decreased to 56.5% in MCF-7 cells, 61.8% in HepG2 and 70.0% in HeLa cells after 96 h at the same treatment concentration. In the case of healthy lymphocytes, it had not demonstrated any cytotoxic effects when treated at the same concentrations (Figure 1(c)).

### Morphological observation

After 48 h of P5 treatment to the cancer cell lines, many morphological changes such as cell rounding up, detachment from the substrate, cell shrinkage, presence of apoptotic bodies along with a decrease in cell concentration were clearly observed in HepG2, HeLa and MCF-7 cells (Figure S1).

### Fluorescence microscopy

**Figure 2.**
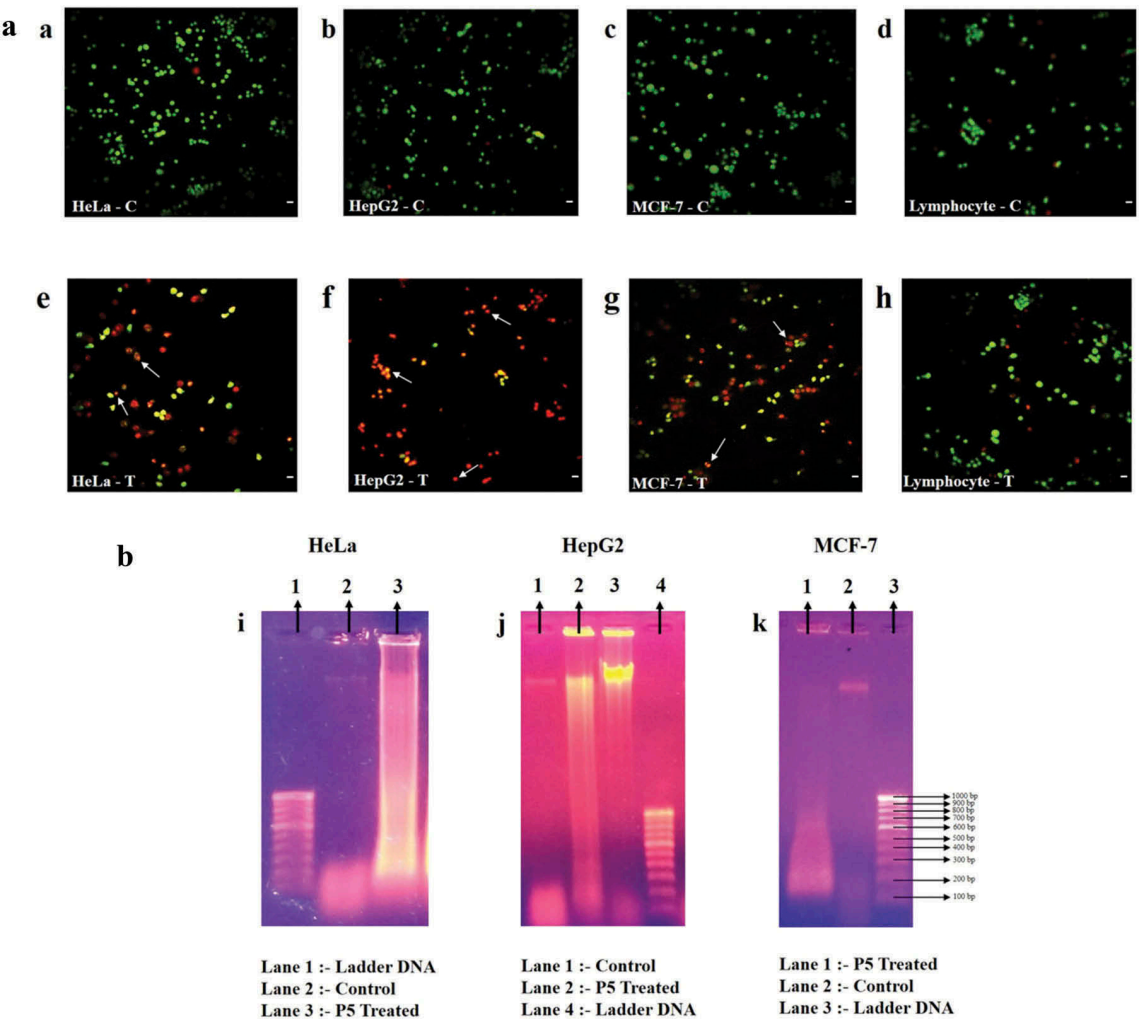
(a). Fluorescence microscopic photographs after AO/EtBr staining. Arrows indicate apoptotic bodies/chromatin condensation. C – control and T – P5 treated; Scale bar: 20 μm. (b): DNA fragmentation analysis of HeLa, HepG2 and MCF-7 cells

After 48 h of P5 treatment, cancer cells were harvested, stained with AO/EtBr. Under the fluorescence microscope, cells fluorescing green (Figure 2a(a–d)) represented the healthy living cells. Cells that fluoresced light orange to deep red (Figure 2a(e–g)) represented apoptotic cells. Figure 2(h) represents the treated lymphocytes, which clearly were fluorescing green, an indication of their viability, just like the untreated lymphocytes. The safety aspect of P5 to normal cells is evident here.

### DNA fragmentation analysis

When the pattern of DNA fragmentation of cancer cell lines treated with P5 for 48 h were analysed, it appeared as a smear in all three cancer cell lines as compared to the intact band in case of untreated control cells (Figure 2b(i–k)).

### LDH activity

**Figure 3.**
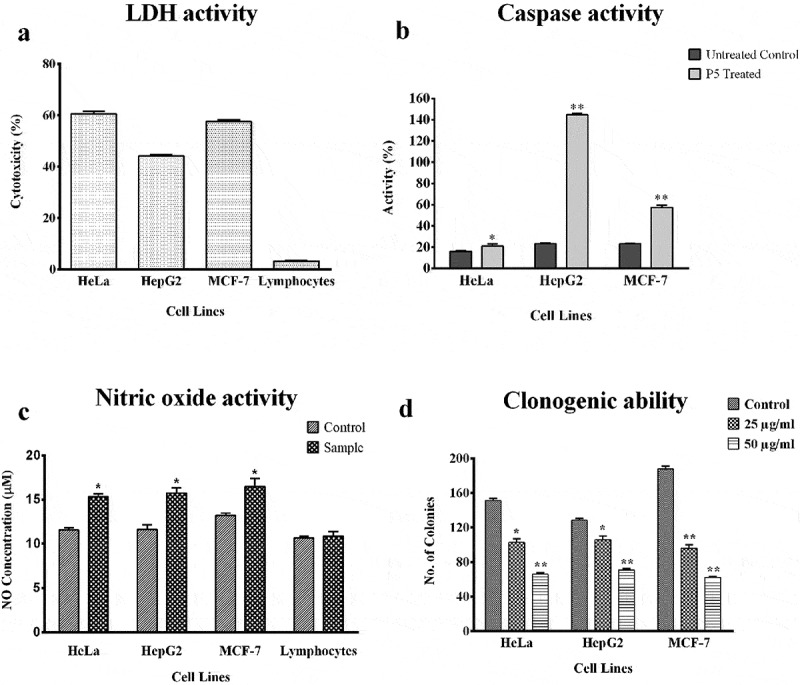
Assays for LDH, Caspase, Nitric oxide and clonogenic ability.*p< 0.05 and **p< 0.01 indicate the levels of significance in comparison to the control

When the LDH activity assay was performed after 48 h of P5 treatment (Figure 3(a)), the highest cytotoxicity was observed against HeLa cells (60.37%) followed by MCF-7 cells with 57.38% and HepG2 cells with 43.29%. A negligible cytotoxicity (2.9%) was observed on the lymphocytes due to P5 treatment.

### Caspase activities

The activities of caspase −3, −7 and −10 were 20.72% in P5 treated HeLa cells as compared to 16.06% in its respective untreated control. A higher activity of 56.84% was observed in P5 treated MCF-7 cells when compared to the 23.15% in the untreated control. The highest caspase activity was observed in HepG2 cells with 144.54% activity after P5 treatment when compared to the 23.68% in the untreated HepG2 cells (Figure 3(b)).

### NO assay

When culture supernatants of the treated cells were evaluated for their NO levels, an increase in NO was observed. The NO release was estimated to be 15.75 µM, 15.33 µM and 16.48 µM as compared to 11.62 µM, 11.58 µM and 13.23 µM of NO in untreated HepG2, HeLa and MCF-7 cell lines, respectively, however, the NO level in lymphocytes after treatment was found to be unaltered (Figure 3(c)).

### Clonogenic assay

Through clonogenic assay, fewer number of colonies of the cancer cells were formed after treatment, which were comparatively smaller in size than the untreated cells (Figure 3(d)). There was a clear reduction in the number of cells present in a single colony and the number of colonies formed due to P5 treatment at 25 and 50 µg/ml concentration. The colony formation potential was dose-dependent. The number of colonies varied between 96-106 at 25 µg/ml concentration and 62–72 at 50 µg/ml concentration of P5, as compared to the 130–190 colonies in the untreated control cells.

### Stages of cell cycle

**Figure 4.**
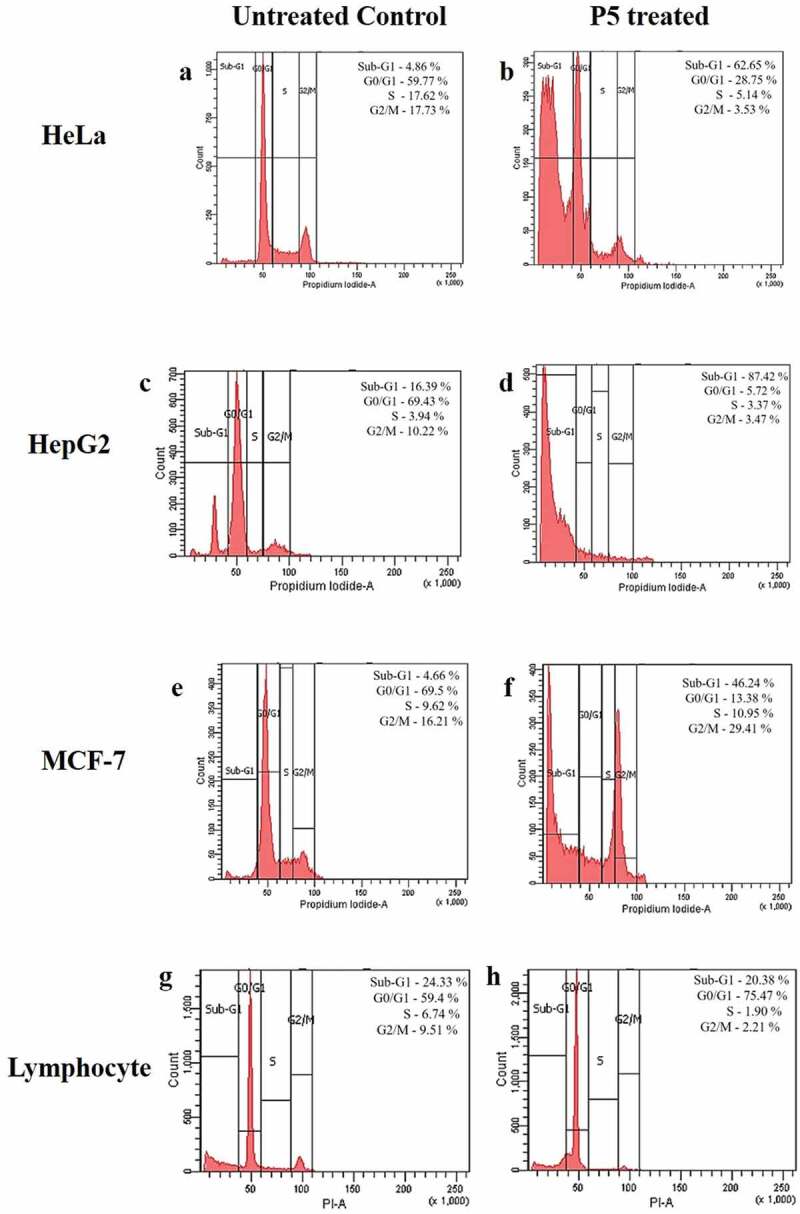
Flow cytometric analysis of cell cycle events after P5 treatment

When the cell cycle of the P5 treated cells was checked through flow cytometry, an increase from 4.86% to 62.65% in the sub-G1 phase and decrease from 59.77% to 28.75% in G0/G1 phase together with a decrease in S and G2/M phase was observed in HeLa cells (Figure 4(a) and b). In HepG2 cells, major accumulation of cells was observed in sub-G1 phase with 87.42% after treatment against 16.39% of cells in controls. The population of cells in G0/G1 phase was found decreased from 69.43% to 5.72% together with a reduction in S and G2/M phase cells (Figure 4 (c,d)). A massive increase in the sub-G1 cells and a decrease in G0/G1, S and G2/M phases of HeLa and HepG2 cells clearly indicated apoptosis of the cells. A similar trend of increased sub-G1 population from 4.66% to 46.24% after treatment was found even in MCF-7 cells along with a decrease in G0/G1 phase with 13.38% cells as opposed to 69.5% cells in the controls. There was accumulation of 29.41% cells in the G2/M phase, suggesting a G2/M phase arrest (Figure 4 (e,f)). Contrary to these results, treatment of P5 to normal lymphocytes resulted in an increased cell concentration together with 20.38% sub-G1 cells in comparison to the 24.33% control cells (Figure 4 (g,h)).

### Bcl-2, Bax and p53 mRNA levels

**Figure 5.**
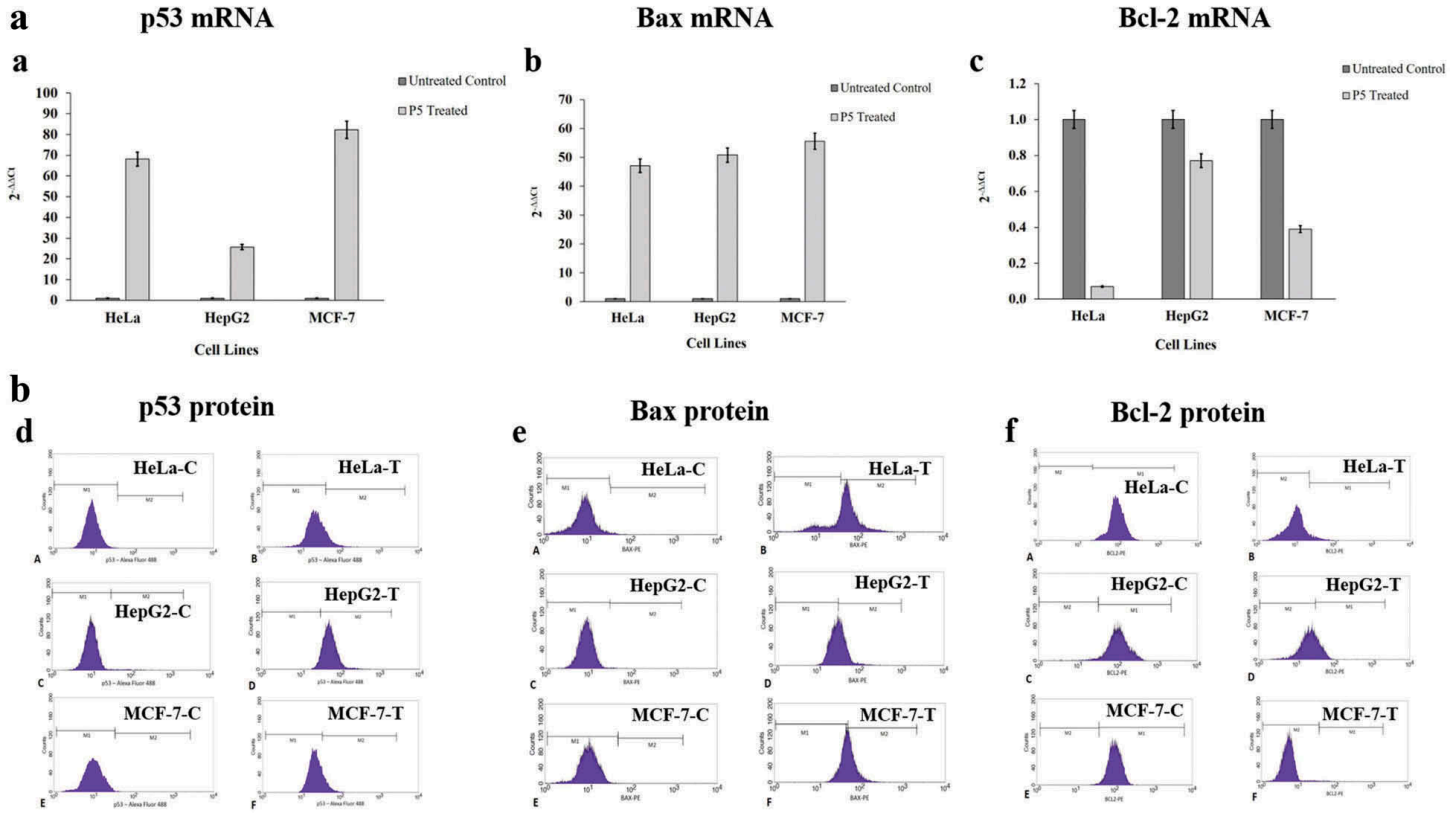
(a). Expression patterns of the *p53, Bax* and *Bcl-2* mRNA in response to P5 treatment. (b). Expression patterns of the *p53, Bax* and *Bcl-2* proteins in response to P5 treatment. C-control and T- P5 treated

The mRNA levels of Bcl-2, Bax and p53were analysed in all three cancer cell lines (Figure 5a(a–c)). The concentration of total RNA isolated from both treated and untreated cell lines ranged from 31.8 to 201.6 ng/μl. After cDNA synthesis with specific primers, the expression levels of p53, Bax and Bcl-2 were determined by RT-qPCR. The relative expression of Bax was increased 47-folds in HeLa, 50.7-folds in HepG2 and 55.5-folds in MCF-7 cell lines after P5 treatment. Simultaneous to this, the expression level of p53 was also higher with a 68.1-folds in HeLa, 25.7-folds in HepG2 and 82.3-folds in MCF-7 cell lines in comparison to their controls. In the same group of treated cells, the expression of Bcl-2 was highly downregulated in HeLa cells corresponding to 14.3-folds decrease followed by 2.6-folds decrease in MCF-7 and 1.3-folds decrease in HepG2 cells as compared to their respective control cells.

### Bcl-2, Bax and p53 protein levels

When the levels of the proteins Bcl-2, Bax and p53were evaluated in the treated cancer cells through flow cytometry (Figure 5b(d–f)), results similar to that observed in RT-qPCR analysis were seen. It was found that the Bcl-2 level was drastically decreased, i.e., from 99.8% to 9.19% in HeLa cells, from 99.35% to 0.77% in MCF-7 cells and from 97.3% to 27.7% in the case of HepG2 cells (Table S1). The findings are significant as it is obvious that the regular cell cycle regulatory mechanism is disrupted in cancer cells with high expression of anti-apoptotic genes, thereby evading death due to apoptosis. It was also seen that Bax expression was upregulated, with an increase from 0.86% of the controls to 73.5% of treated HeLa cells expressing it. The expression of Bax in HepG2 cells increased from 0.33% in control cells to 48.6% and in MCF-7 cells, it went up from 0.03% to 48.72% after 48 h of P5 treatment (Table S2). Along with these two, the expression of p53 was also greatly enhanced in the case of HepG2 cells with an increase from 1.25% to 93.16%, in HeLa from 0.14% to 16.4% and in MCF-7 cells from 0.36% to 17.5% (Table S3).

### Characterisation of P5 through GC-MS analysis

**Figure 6.**
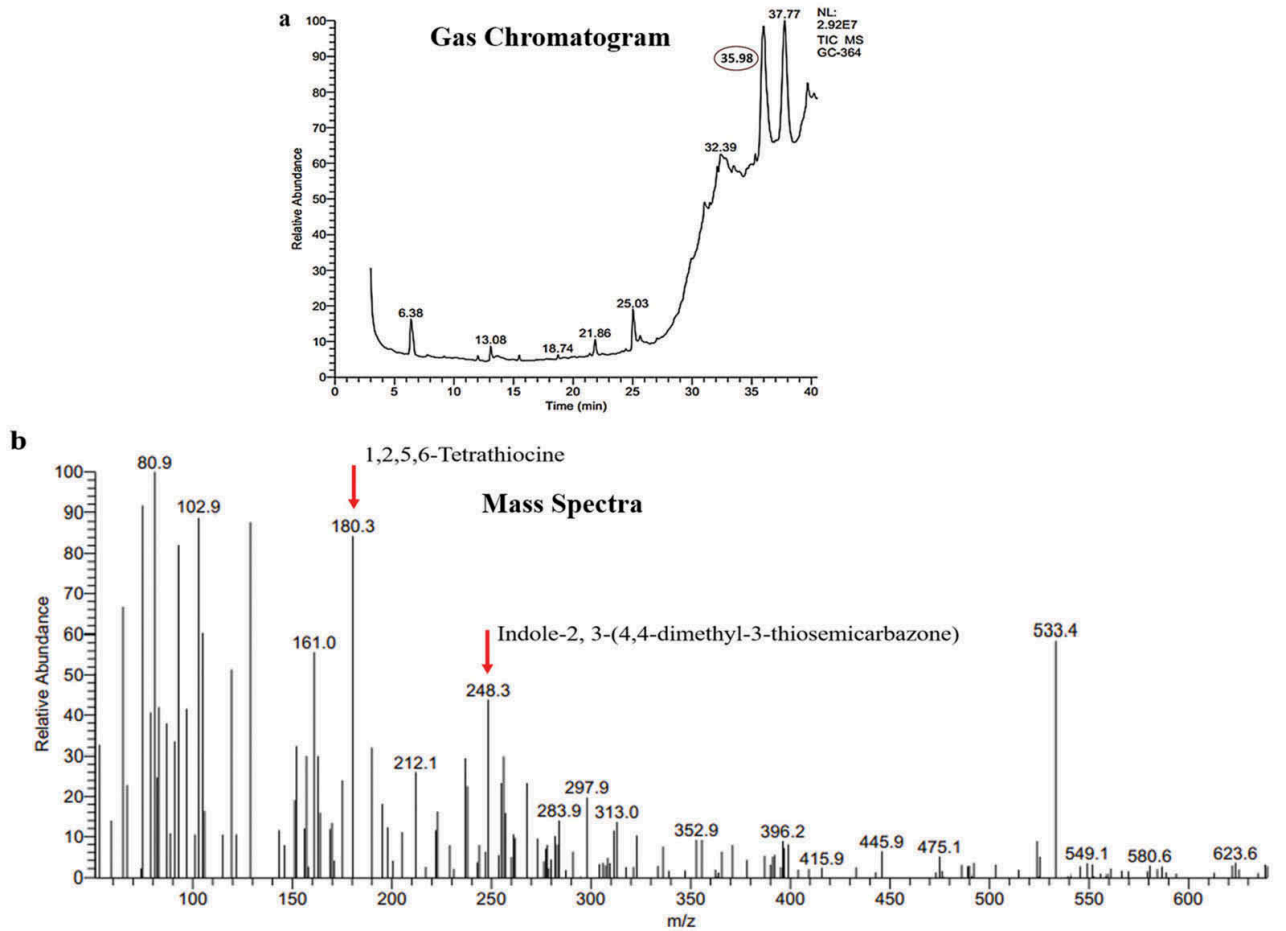
GC-MS analysis of P5 fraction

The GC-MS analysis of P5 resulted in five major peaks at 6.38, 25.03, 32.37, 35.98 and 37.77-min retention time (Figure 6(a)). A thorough library search from the cancer resource database (http://data-analysis.charite.de/care/) indicated compounds such as1,2,5,6-Tetrathiocine with an *m/z* ratio of 180.3 and Indole-2, 3-(4,4-dimethyl-3-thiosemicarbazone), an indole-thiosemicarbazone derivative with an *m/z* ratio of 248.3 (Figure 6(b)) from the fraction at the retention time 35.98 min. Among these, Indole-2, 3-(4,4-dimethyl-3-thiosemicarbazone) has been reported to have anti-cancer activity. It has been isolated earlier from plant sources and also from *Bufo* frogs but not from *Penicillium rubens* (Ibrahim and Elsaman 2018).

## Discussion

Cancer is a disease that efficiently overcomes all kinds of available therapies and the side effects of the existing therapeutic options are far more harmful than the disease itself. Finding an alternative therapy is of utmost importance in cancer management. Filamentous fungi, especially of the genus *Penicillium*, have contributed immensely towards research in industrial and pharmaceutical domains with several lead compounds towards cancer therapy being reported from various species of *Penicillium*. But till now no anti-cancer drug from *Penicillium* sp. has been approved by FDA and reached the consumer market. In the present study, a newly isolated strain of *P. rubens* had demonstrated promising anti-cancer properties to *in-vitro* cancer cell lines. Though there are reports of secondary metabolites with antitumor activity from *Penicillium* sp. (Bladt et al. 2013; Chavez et al. 2015; Nicoletti and Trincone 2016; Feng et al. 2018; Farha and Hatha 2019; Orfali and Perveen 2019), a metabolite from *P. rubens* having promising anti-cancer properties has been reported for the first time in this present work.

In the current study, the bioactive fraction (P5) obtained from the partial purification of the methanol extract from *P. rubens* JGIPR9 was tested for its anti-cancer activity on HepG2, HeLa and MCF-7 cell lines. The IC_50_ value of the fraction was 24.4 µg/ml for HepG2, 11.0 µg/ml for HeLa and 16.0 µg/ml for MCF-7 cells. These IC_50_ values are well within the range suggested by the FDA. These values are better than that reported earlier about the IC_50_ value of fudecadione A, isolated from *Penicillium* sp. BCC 17,468 (Pittayakhajonwut et al. 2011). The cytotoxic effect of P5 on cancer cells was found to be significantly higher than the positive control (tamoxifen) used in the study. The cytotoxic effect of P5 was first confirmed by observations under both inverted and fluorescence microscopes, which revealed the presence of fragmented particles, apoptotic bodies and reduced cell numbers which are considered as hallmarks of apoptosis.

Evidence for the direct cytotoxicity of P5 was provided by the results of LDH cytotoxicity assay. Loss of membrane integrity due to the cytotoxic effects by external agents causes LDH to release to the cytoplasm. Here, in the present study, after P5 treatment, the percentage cytotoxicity was high in HeLa, HepG2 and MCF-7 cells which might be one of the triggering factors that led to cell death observed in this case.

To be an efficient anti-cancer agent, a compound needs to be having anti-proliferative effects too on the cancer cells apart from exerting direct cytotoxic effects. Proof for anti-proliferative property of P5 was given by the results of clonogenic assay, which resulted in a reduction (dose-dependent) in the number of colonies formed due to its treatment to the cancer cells.

One of the major characteristics for an ideal drug for cancer therapy is its efficiency to inhibit cell proliferation and induce apoptosis in cancer cells. The ability of P5 to induce apoptosis in the treated cells was checked by analysing the DNA fragmentation pattern, as it is a clear indicator of apoptosis happening in the cells. In the present study, the DNA of treated cancer cells appeared like a smear upon electrophoresis, indicating higher degree of apoptosis in these cells (Paul et al. 2010; Mathi et al. 2014).

During DNA fragmentation, elevation of caspase enzymes in cells is evident, as caspases play crucial roles in the apoptotic machinery. The compound P5 in the present study was inducing higher caspase activities in the cancer cell lines, thereby providing proof for its apoptogenic property.

Bcl family of proteins are important regulators of apoptosis, where Bcl-2 and Bax are the proteins responsible for inhibiting and promoting apoptosis, respectively. In addition to this, the tumour suppressor p53 plays a significant role in controlling the cell cycle progression along with inducing apoptosis (Basu and Haldar 1998). In our study, as per the results of RT-qPCR analysis, the mRNA levels of Bax and p53 were markedly increased, concomitant to a decrease in Bcl-2 in the P5-treated cancer cells, but their levels of expression varied slightly among these cells, which might be due to the cell line specificity of P5. Flow cytometry results indicated a similar trend of Bax, p53 protein upregulation and Bcl-2 protein suppression. When these results were put together, we could observe that the co-ordinated effects of the upregulated Bax and p53 might be the driving force behind the observed apoptosis in the cancer cells. Though slight discrepancy was seen in the quantities of the mRNAs and proteins, we can say that this could be due to the differences in the regulation of transcription and translation after P5 treatment (Maier et al. 2009; de Sousa et al. 2009; Liu et al. 2016).

Cell cycle analysis indicated an activation-induced cell death or direct apoptosis is suggestive for HeLa and HepG2 cells treated with P5 fraction, as an increase in the sub-G1 phase cells was observed. In P5 treated MCF-7 cells, G2/M phase arrest was noticed. A similar trend of increased apoptotic cells, i.e., sub-G1 phase was earlier reported due to the addition of bioactive compounds on cancer cell lines (Kajstura et al. 2007; Belayachi et al. 2017). A G2/M phase arrest in combination with an increase in the sub-G1 population has been reported earlier (Burgess et al. 2006; da Silva et al. 2015). Though P5 treatment resulted in increased sub-G1 phase and decreased cell count in cancer cells, the same did not cause any significant changes to the cell cycle of normal lymphocytes, clearly reflecting the specificity of P5 to the cancer cells.

Another indicator of apoptosis is the NO release from cells upon drug treatment. NO is synthesised by three distinguishable forms of nitric oxide synthase (NOS) amongst which iNOS (inducible NOS) is responsible for apoptosis induction, oxidative stress and DNA damage (Martin et al. 1999). In the current study, there was an increase in the NO levels due to P5 treatment which was simultaneous to the apoptosis of the cells indicating the activation of iNOS. This increased NO level might also be contributing to the observed cell death in cancer cells. According to an earlier report, increased level of NOS was the major cause for p53 accumulation, cell cycle arrest and apoptosis (Hsieh et al. 1999).

The GC-MS analysis of P5 fraction, indicated the presence of an indole-thiosemicarbazone derivative, with many reports indicating its anti-cancer properties. The compound has been isolated from plants belonging to *Isatis* genus, fruits from the cannon ball tree *Couroupita guinanensis*Aubl. and *Calanthe discolour* Lindl., and was also found to be secreted by the parotid gland of *Bufo* frogs. The anti-cancer activity of these derivatives has been well documented (Hall et al. 2009; Hussein et al. 2015; Ibrahim and Elsaman 2018). It can be assumed that the presence of this compound might be responsible for the observed anti-cancer property of P5 in the current study.

To be an ideal anti-cancer agent, it is necessary that the selected compound needs to be effective not only against the cancer cell proliferation, but also should be safe to the normal healthy cells. To verify the non-toxicity of this promising compound, it was checked on normal human peripheral lymphocytes and it was found that P5 fraction was non-toxic to the lymphocytes at all the tested concentrations. When the total cell count was taken through trypan blue assay (Table S4), no significant changes were found. The LDH assay results further confirmed the non-toxicity, as there was negligible damage to the lymphocyte membrane, thereby indicating its safety to the normal cells.

As many of the natural anti-cancer compounds in clinical use today are associated with undesirable side effects, finding an alternative compound with least toxicities will be of great significance. The fraction P5 from *Penicillium rubens* JGIPR9 in the current study offers such a promise towards drug development.

## Supplementary Material

Supplemental Material

Supplemental Material

## Data Availability

The data that support the findings of this study are available from the corresponding author (VKN), upon reasonable request.
