# Supplementary figures and images for: Modulation of Bax and Bcl-2 genes by secondary metabolites produced by Penicillium rubens JGIPR9 causes the apoptosis of cancer cell lines
Source: Mycology. 2019 Dec 26;12(2):69–81. doi: 10.1080/21501203.2019.1707315 (PMC8128197; doi:10.1080/21501203.2019.1707315)

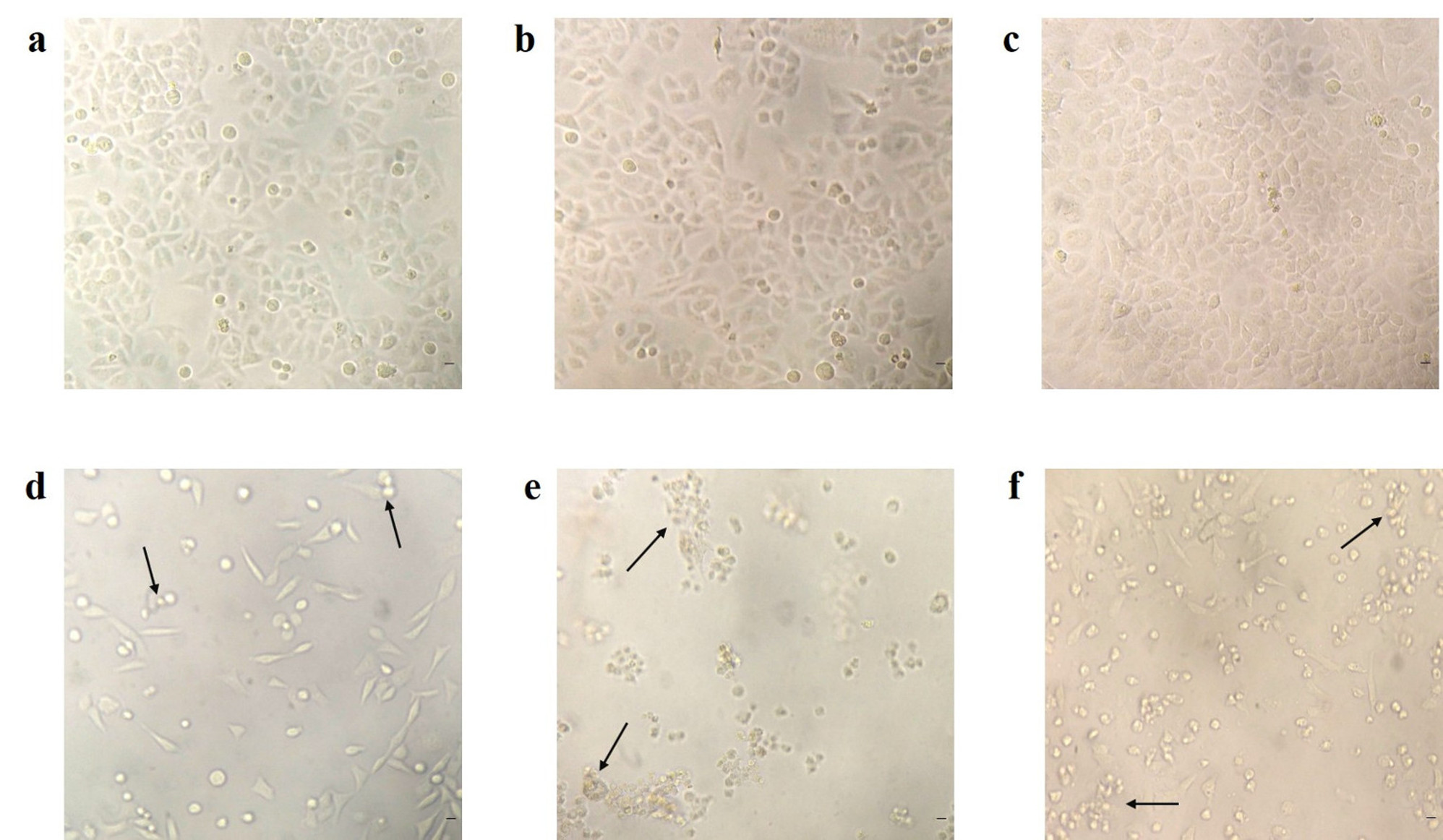

Supplement: Supplemental Material [file TMYC_A_1707315_SM1877.jpg]
