# Supplementary material for: Modulation of Bax and Bcl-2 genes by secondary metabolites produced by Penicillium rubens JGIPR9 causes the apoptosis of cancer cell lines
Source: Mycology. 2019 Dec 26;12(2):69–81. doi: 10.1080/21501203.2019.1707315 (PMC8128197; doi:10.1080/21501203.2019.1707315)
Supplement: Supplemental Material [file TMYC_A_1707315_SM1876.docx]

**Supplementary material**

**Modulation of Bax and Bcl-2 genes by secondary metabolites produced by *Penicillium rubens* JGIPR9 causes the apoptosis of cancer cell lines**

**Prerana Venkatachalam^a^ and Varalakshmi Kilingar Nadumane^a^***

^a^Department of Biotechnology, School of Sciences, JAIN (Deemed-to-be University), 18/3, 9^th^ main, 3^rd^ block, Jayanagar, Bengaluru - 560 011, India

*****Corresponding author - Varalakshmi Kilingar Nadumane,

e-mail id:[kn.varalakshmi@jainuniversity.ac.in](mailto:kn.varalakshmi@jainuniversity.ac.in)


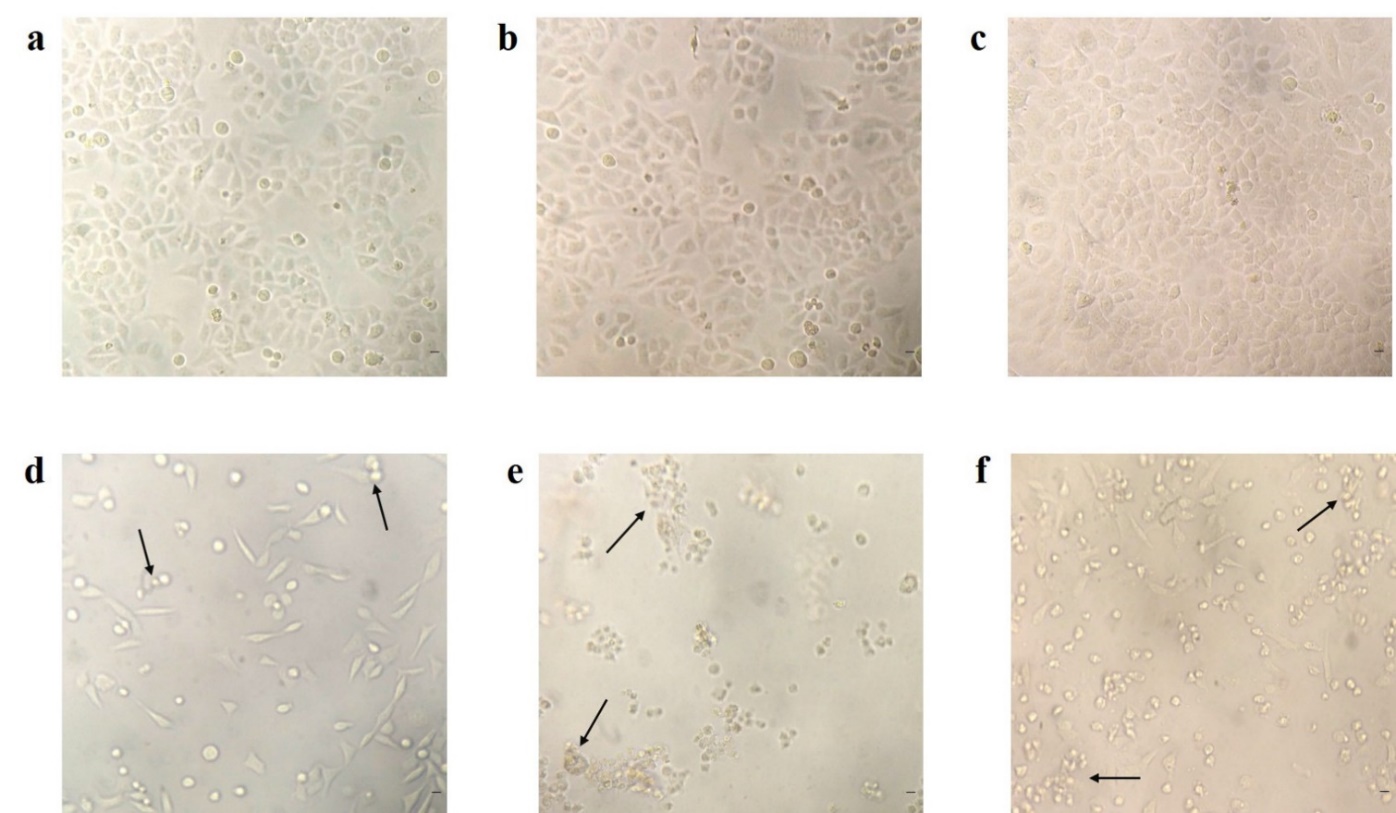


**Figure S1** Morphological changes observed in cancer cells after P5 treatment for 48 h. (a) untreated HeLa, (b) untreated HepG2, (c) untreated MCF-7, (d) treated HeLa, (e) treated HepG2 and (f) treated MCF-7. The arrows correspond to the representative apoptotic cells. Scale bar: 20 μm

**Gene expression by flow cytometry**

The histograms obtained are being represented by M1 and M2 populations. Positive expression (increased fluorescence) due to treatment is indicated by the shift of the fluorescence peak towards the right in comparison to the control. Shift of the peak towards left indicates a negative expression (decreased fluorescence) in comparison to the control.

**Table S1** Percentage of cell population expressing the anti-apoptotic Bcl-2.

| Cell Line | HeLa | | HepG2 | | MCF-7 | |
| --- | --- | --- | --- | --- | --- | --- |
| % cell population | M1 | M2 | M1 | M2 | M1 | M2 |
| Control | 99.81 | 0.20 | 97.33 | 2.74 | 99.35 | 0.67 |
| Treated | 9.19 | 91.35 | 27.77 | 72.85 | 0.77 | 99.20 |

The M1 and M2 populations in control cells represent the positive and negative expression respectively of Bcl-2. In treated cells, M1 population represents the positive expression and M2 population represents the downregulation of the anti-apoptotic Bcl-2 gene.

**Table S2** Percentage of cell population expressing the pro-apoptotic Bax.

| Cell Line | HeLa | | HepG2 | | MCF-7 | |
| --- | --- | --- | --- | --- | --- | --- |
| % cell population | M1 | M2 | M1 | M2 | M1 | M2 |
| Control | 98.40 | 0.86 | 99.68 | 0.33 | 99.96 | 0.03 |
| Treated | 26.42 | 73.51 | 52.51 | 48.61 | 52.29 | 48.72 |

The M1 and M2 populations in control cells represent the negative and positive expression respectively of Bax. In treated cells, M1 population represents the negative expression and M2 population represents the upregulation of the pro-apoptotic Bax gene.

**Table S3** Percentage of cell population expressing the tumour suppressor p53.

| Cell Line | HeLa | | HepG2 | | MCF-7 | |
| --- | --- | --- | --- | --- | --- | --- |
| % cell population | M1 | M2 | M1 | M2 | M1 | M2 |
| Control | 99.86 | 0.14 | 98.77 | 1.25 | 99.66 | 0.36 |
| Treated | 83.95 | 16.42 | 7.21 | 93.16 | 82.99 | 17.49 |

The M1 and M2 populations in control cells represent the negative and positive expression respectively of p53. In treated cells, M1 population represent the negative expression and M2 population represent the overexpression of the p53 gene.

**Table S4** Trypan blue assay

| Cell Line | Untreated Control | | P5 Treated | |
| --- | --- | --- | --- | --- |
|  | Viable cell count (1x10^6^ cells/ml) | % Viability | Viable cell count (1x10^6^ cells/ml) | % Viability |
| HeLa | 4.0 | 98.14 | 2.9 | 59.6 |
| HepG2 | 4.8 | 96.2 | 3.5 | 64.5 |
| MCF-7 | 3.2 | 97.7 | 1.3 | 54.97 |
| Lymphocytes | 2.2 | 96.5 | 2.1 | 95.27 |
|  |  |  |  |  |

Cell viability being assessed by trypan blue dye exclusion method revealed that there was a reduction in the viability along with reduction in cell number in the P5 treated cancer cells. On the contrary, it was found that the cell number and viability were not much altered in the case of normal lymphocytes due to P5 treatment.
